# Take Time by the Forelock

**Published:** 1875-01

**Authors:** 


Take Time by the Forelock — Whatever else you are behind in, don't delay to store your fuel in the cellar for the cold season until a storm comes. If you have place for it apart from the dwelling, it won't make so much difference, but even then you will have to pay more for it, besides suffering from the annoyance of receiving it in an inclement season. But it is really dangerous to house coal in the cellar when it is wet with snow or rain. Some people sprinkle coal when they put it in, in order to avoid dust. They may not consider that when they store damp fuel, they also store up sore throats and numerous other ills. Such is the fact. Even the fire-damp which escapes from coal mines arises from the slow decomposition of moist coal at temperatures but little above that of the atmosphere, but under augmented pressure. By wetting a mass of freshly-broken coal, and putting it in a warm cellar, the mass is heated to such a degree that carburetted and sulphuretted hydrogen are given off for long periods of time, and pervade the whole house. The liability of wet coal to mischievous results, under such circumstances, may be appreciated from the circumstance that there are several instances on record of spontaneous combustion of wet coal when stowed into the bunkers or holds of vessels. And from this cause, doubtless, many missing coal vessels have perished. Wet wood is less harmful, but even that gives off very unwholesome and dangerous exhalations.

---

The Breath.—Of this there are two kinds—the breath we take in, which is, or ought to be, pure air, composed, on the whole, of oxygen and nitrogen, with a minute portion of carbonic acid —and the breath we give out, which is an impure air, to which has been added, among other matters which will not support life, an excess of carbonic acid. This carbonic acid gas, when warm, is lighter than the air, and ascends; and, when at the same temperature as common air, is heavier than that air, and descends, lying along the floor, just as it lies often in the bottom of old wells or old brewers' vats, as a stratum of poison, killing occasionally the men who descend into it. Hence a word of admonition is addressed to those who think nothing of sleeping on the floor; and hence, as the poor in great cities are too apt, in times of distress, to pawn their bedsteads and keep their beds, the friends of the poor—those who go about doing charitable work among them at this season—are entreated never to let this happen, and to implore them to keep the bedstead, whatever else may go, to save the sleeper from the carbonic acid stratum which lies close on the floor, especially in cold weather.

---

The Hour of Death.—A great majority of all deaths occur before noon. Deaths from chronic diseases are more numerous between the hours of 8 and 10 in the morning than any other time of the day, while they are fewest between the hours of 8 and 10 in the evening. In the case of acute diseases, such as continued fevers, pneumonia, etc., the largest number of deaths take place either in the early morning, when the powers of life are at their lowest, or in the afternoon, when acute disease is most active.

